# Practical rhythm: integrating sound and attunement into psychiatric care

**DOI:** 10.1192/bjb.2025.10208

**Published:** 2026-04

**Authors:** Hesham Y. Elnazer

**Affiliations:** DM, CCT, MBBCh, FRSA, FRCPsych, Consultant Neuropsychiatrist, Department of Neuropsychiatry, Priory Wellbeing Centre, Southampton, UK; Senior Clinical Lecturer, Department of Psychiatry, Brighton and Sussex Medical School, Brighton, UK

Psychiatry is a discipline of listening, yet our clinical listening often privileges speech while neglecting the wider sonic environment in which patients live and recover. Across cultures, ritual chant, lullaby and birdsong functioned as regulators of arousal and social belonging long before modern neuroscience explained the mechanisms involved. Auditory signals travel from the cochlea through brainstem and thalamic relays to the auditory cortex but crucially also project rapidly to limbic structures (amygdala, hippocampus) and reward networks (ventral striatum).^1^ Music and rhythm therefore access affective and motivational circuits within milliseconds; rhythm perception couples sensory prediction with motor systems and dopaminergic anticipation, providing an embodied route to attention and mood modulation.^2^

Clinical work already leverages these properties. Music therapy trials have reported modest but consistent reductions in anxiety and depressive symptoms when used alongside standard treatment.^3^ In procedural or critical care settings, tailored music reduces physiological stress markers such as heart rate and systolic blood pressure.^4^ For patients with impaired language (dementia, acquired brain injury), rhythm and song may provide a direct pathway to memory and social engagement.^5^

Digital sound practices, notably binaural beats, an auditory illusion created by presenting slightly different tones to each ear, inhabit a different epistemic space. Enthusiasts report benefits for focus and relaxation, and such recordings have become culturally prominent on streaming platforms. Some meta-analyses show small, heterogeneous effects on anxiety and cognition; however, electroencephalogram evidence for reliable cortical entrainment remains inconsistent.^6,7^ Thus, the evidence is not yet sufficient to endorse binaural beats as a treatment, but their popularity signals a contemporary re-enactment of an ancient impulse: using patterned sound to regulate mind and sociality.

What matters for practising psychiatrists, therefore, is less whether rhythm is labelled as ‘therapy’ and more how this awareness changes our practice. The therapeutic encounter itself has a tempo, defined by the pacing of speech, the timing of silence and even the synchrony of breath. Neuroscience suggests that this is more than metaphor; neural synchrony between individuals is correlated with empathy and cooperation, making temporal attunement a cultivable clinical skill.^8^

I therefore propose three steps for clinicians and services. First, we can prescribe the sound environment. This could involve co-creating patient-selected playlists for anxiety or agitation, or using quiet, natural soundscapes to calm in-patient ward environments. Second, we should adopt a curious but critical stance towards digital sound tools such as binaural beats. We can note patient demand, discuss the limited but evolving evidence and encourage self-monitoring of effects, without overclaiming. Third, and most fundamentally, we must cultivate ‘listening as tempo’ as a core skill. This means training ourselves and our teams to be attuned to the rhythmic dimensions of interaction, knowing when to slow a conversation, when to use silence and how to align with a patient’s emotional pace.

By reclaiming psychiatry’s auditory heritage in these practical ways, we can augment both empathy and physiological regulation, bridging the gaps between biology, culture and daily care.
